# Arsenic and Mercury Containing Traditional Chinese Medicine (Realgar and Cinnabar) Strongly Inhibit Organic Anion Transporters, Oat1 and Oat3,* In Vivo* in Mice

**DOI:** 10.1155/2015/863971

**Published:** 2015-12-16

**Authors:** Wen-Hao Yu, Na Zhang, Jin-Feng Qi, Chen Sun, Yong-Hui Wang, Mei Lin

**Affiliations:** ^1^Department of Pharmacology, Guangzhou University of Chinese Medicine, Guangzhou 510006, China; ^2^Pharmacy Department, Zhumadian People's Hospital, Zhumadian, Henan 463000, China; ^3^Pharmacy Department, Nanhai Hospital of Chinese Medicine, Foshan, Guangdong 528200, China

## Abstract

Toxic heavy metals, including mercury (Hg) and arsenic (As), accumulate preferentially in kidneys and always cause acute renal failure. The aim of this study was to investigate whether these samples affect organic anion transporters, Oat1 and Oat3,* in vivo* in mice kidney. Mice (*n* = 10) were orally treated with investigational samples. After last administration, all mice were i.v.* p*-aminohippuric acid (PAH), and the blood and kidneys samples were collected. The concentrations of PAH were quantified by spectrophotometry. mRNA expressions of Oat1 and Oat3 were assayed by real-time PCR. In comparison with corresponding control, major pharmacokinetic parameters of PAH in sera were significantly changed by investigational samples (*p* < 0.05), PAH accumulations in the kidney tissues were significantly higher (*p* < 0.05), PAH uptake by renal slices was greatly reduced, Oat1 and Oat3 mRNA expression were significantly inhibited in investigational sample groups. Arsenic and mercury containing traditional Chinese medicine (Realgar and Cinnabar) probably induce kidney damage through inhibiting several members of the organic anion transporters (such as OAT1 and OAT3).

## 1. Introduction

Drug transporters are known to have a significant impact on the absorption, distribution, elimination, and toxicity of a large number of drugs [1]. It is noteworthy that organic anion transporters (OATs) which are the members of Solute Carrier Family 22 (SLC22) play a pivotal role in renal excretion of water-soluble or negatively charged organic compounds (including endogenous waste products, numerous drugs) and their metabolites. However, some of them lead in some cases to nephrotoxicity [1, 2]. A substantial fraction of such compounds carries a net negative charge at physiological pH and hence is referred to as organic anions (OAs).* p*-Aminohippuric acid (PAH) which is known to interact with multiple basolateral transporters in proximal tubule cells is the prototypic substrate for what is frequently referred to as the “classic” process of renal organic anion secretion [3]. More than a decade ago, two PAH-transporters [organic anion transporter (OAT1 and OAT3)] were identified and functionally characterized on the molecular level [4, 5]. In mice, Oat1 (*Slc22a6*) is detected exclusively in the proximal tubules; however Oat3 (*Slc22a8*) is localized in the proximal tubule, cortical, and medullary thick ascending limb of Henle's loop, connecting tubules, and cortical and medullary collecting ducts [6]. The overlapping substrate specificity and localization at the basolateral membrane of proximal tubules from Oat1 and Oat3 support the assumption that both transporters may play a principle role in the absorption of PAH and other OAs [5, 7].

Toxic heavy metals, including mercury (Hg) and arsenic (As), accumulate preferentially in kidneys and always cause acute renal failure [8]. Renal proximal tubular cells represent the major target site where highly reactive mercuric ions are proved to rapidly accumulate and induce cell injury [9]. However, both Realgar and Cinnabar are included in some prescription of Chinese herbal formulae. The main component of Realgar is As_2_S_2_ and that of Cinnabar is HgS. Although As_2_S_2_ and HgS are difficult to be adsorbed by the gastrointestinal tract of mammals, they slightly contain soluble and extremely toxic components, arsenic (As^3+^) and mercury (Hg^2+^), such as HgCl_2_ and As_2_O_3_ [10]. As^3+^ and Hg^2+^ can easily gain access to proximal tubular cells primarily via Oat1 and Oat3 in the basolateral membrane [11]. To our knowledge, there is some information regarding modifications (inhibition) of these transporters in nephrotoxic acute renal failure [12, 13]. No similar study has conducted the interaction between arsenicand mercury containing traditional Chinese medicine (Realgar, Cinnabar, and HgCl_2_) with organic anion transporters in mammalian kidney in literature. Therefore, the aim of the present study is to evaluate the expression and function of Oat1 and Oat3 after administering arsenic and mercury containing traditional Chinese medicine (Realgar and Cinnabar)* in vivo* in mice.

## 2. Materials and Methods

### 2.1. Chemicals and Reagents

Realgar and Cinnabar were purchased from Guangzhou Pharmaceuticals Corporation (Lot. 120612). HgCl_2_ was purchased from Guangzhou Chemical Reagent Factory (Lot. 110601).* p*-Aminohippuric acid (98%, Aladdin, Lot. 120816); probenecid (Aladdin, Lot. 130512); sodium carboxymethylcellulose (CMC-Na, Sinopharm Chemical Reagent Beijing Co., Ltd., China, Lot. 111015); Trizol, RT reagent Kit, and SYBR Premix Ex Taq II (Lot. D9108A, Lot. DRR037A, and Lot. DRR081A, Takara, Dalian, China); and the primers for Oat1, Oat3, and GAPDH were synthesized by Sangon Biotech Co., Ltd., (Shanghai, China). Other chemicals used were of analytical grade commercially available. 

### 2.2. Preparation of Investigational Samples

Four investigational samples (Realgar, levigated Realgar, Cinnabars, and HgCl_2_) were made into suspension by 0.5% CMC-Na. Grinding Realgar was prepared freshly according to the Chinese Pharmacopeia. To assure quality control, the Cinnabar was validated by the method of the Chinese Pharmacopeia (China Pharmacopoeia Committee, 2010). In order to examine tiny amount water soluble mercury (Hg^2+^) in Cinnabar, the method of Huo et al. [14] was adopted [(artificial gastric juice, containing 0.08 N HCl (pH 1.5) and 1% pepsin (Sino-American Biotechnology Co., Ltd., USA, Lot. 140218)], and its content was detected by flame atomic absorption spectrophotometer (AAS) (Thermo, USA) [15].

### 2.3. Experimental Animals and Treatment Protocol

This study was carried out using adult NIH mice (certificate number SCXK 2013-0020), weighing 20~25 g sourced from the Experimental Animal Centre of Guangzhou University of Chinese Medicine. The animal experimental procedures were approved by the animal Ethics Committee of Guangzhou University of Chinese Medicine, Guangzhou, China. All mice were given standard mice chows and pure water ad libitum and housed at 20~26°C. Eleven groups of mice (50% for each gender) were orally given pure water (water control group), 0.5% CMC-Na solution (solvent group), and probenecid (50 mg/kg, positive water control group), and investigational samples were orally given at two (high and low) dosages: Realgar (60 mg/kg and 15 mg/kg), levigated Realgar (60 mg/kg and 15 mg/kg), Cinnabar (120 mg/kg and 30 mg/kg), and HgCl_2_ (0.2 mg/kg and 0.05 mg/kg), which were twice a day for continuous five days.

### 2.4. Blood Sampling and Kidney Removal

On the experiment day, 60 min after the last administration of investigational samples, all mice were single i.v. PAH (30 mg/kg B.W., aqueous solution), respectively, according to the protocol of Bertani et al. [16] and blood samples were collected from 10 mice/group by euthanization in each time point at 1.0, 2.5, 5.0, 7.5, 10.0, 20.0, and 30.0 min after PAH injection. Blood samples were centrifuged at 2000 ×g for 5 min. The obtained sera were stored at −20°C until measurement. The two kidneys were rapidly removed and stored at −80°C for later assays.

### 2.5. PAH Pharmacokinetic Studies

These studies were done in a manner similar to the literatures by Brandoni et al. and Cerrutti et al. [17, 18]. The concentration of PAH in sera was determined according to Saikan and Kiguchi [19] method. The sera concentration versus time curve for PAH, for each individual animal, was fitted with PK software DAS 2.0. The data were fitted to a biexponential curve. The choice of the best fit was based on the determination of coefficient values (*R* ^2^) and LSD test [20]. All fits should have *R* ^2^ values > 0.98. The following equation was used to describe the biexponential concentration-time curves:

(1) $$\begin{array}{c} C_{p}=A\cdot e^{-\alpha \cdot t}+B\cdot e^{-\beta \cdot t}, \end{array}$$

where *C*_*p*_ is PAH serum concentration (mg/mL) at time *t* (min) after administration: constant *α* presents the distribution from the central compartment, and *β* presents an equilibrium constant reflecting the dynamics between *k*_21_ and *k*_10_. *A* and *B* represent the initial values of the distribution and elimination components, respectively, extrapolated from *y*-axis intercept. The estimate parameters (*α*, *β*, *A*, and *B*) were used to solve the first-order rate constants of transfer from the central to peripheral compartments (*k*_12_, *k*_21_) and the elimination rate constant from the central compartment (*k*_10_) with classical equations. Derived parameters, elimination half-life (*t*_1/2*β*_), total volume of distribution (Vd_T_), total clearance (CL_T_), and area under the curve (AUC_0–30 min_), were calculated according to standard procedures for the compartmental analysis. Concentration of PAH in serum was measured using the method described by Di Giusto et al. [21].

### 2.6. Accumulations of PAH to Kidney

The mice renal homogenate was prepared by Shihana et al. [22] protocol. Each right kidney was cut into small pieces, which were put into a glass homogenizing tube containing PBS (200 mM sucrose, 137 mM NaCl, 2.7 mM KCl, 10 mM Na_2_HPO_4_, and 2 mM NaH_2_PO_4_; pH 7.4) at a ratio of 8 mL/g wet weight and then were homogenized with a motor-driven Teflon pestle (1000 rpm/min) and spun down for 30 min at 20,000 ×g. The supernatant was isolated and then stored at −20°C until determination. The kidney homogenates were prepared from each group which was removed at 1, 5, 10, 20, and 30 min after i.v. PAH. Protein content of kidney homogenates was quantified using the Lowry Folin phenol reagent (Nanjing Jiancheng Bioengineering Institute, Ltd., China, Lot. 130918). The concentrations of PAH in kidney homogenate were determined the same as that in the sera.

### 2.7. PAH Uptake by Mice Renal Slices

The uptake of PAH by mice renal slices was investigated using the procedures described by Henderson and Lindup [23]. Briefly, mice were executed, and the kidneys were immediately harvested, decapsulated, and placed in an ice-cold oxygenated rinse PBS (pH 7.4). Renal slices (weight 10–20 mg/slice) were cut freehand with Gillette valet strip blades to about 0.2 inches in length (Sabre International Products Ltd., Reading, UK). Slices of one kidney were preincubated for 5 min at 37°C and incubated in a 12-well plate with 1 mL of oxygenated PAH-buffer which consisted of 2 mM PAH, 97 mM NaCl, 40 mM KCl, 0.74 mM CaCl_2_, and 7.5 mM sodium phosphate-chloride buffer in each well. The uptake study was carried out at 37°C in the shaking bath. After incubating for 20 min, each slice was rapidly removed from the PAH-buffer, immediately inactivated the proteins with trichloroacetic acid, was washed in ice-cold saline, was blotted on filter paper, was weighed, and then was homogenized at a ratio of 3 mL/g wet weight. The following operations were the same as described in Section 2.6.

### 2.8. RNA Isolation and Real-Time PCR

Total RNA was extracted using Trizol reagent from renal cortical tissues according to the manufacturer's instructions and RNA concentration and purity were evaluated by measuring the ratio of A_260 nm_/A_280 nm_. First-strand cDNA was generated by adding 1 *μ*g total RNA; 2 *μ*L 5x gDNA Eraser Buffer; 1 *μ*L gDNA Eraser; 5 *μ*L RNase Free dH_2_O (step 1: 42°C, 2 min for genomic DNA elimination reaction); 2 *μ*L 5x PrimeScript Buffer 2; 1 *μ*L Prime Script RT Enzyme Mix I; 1 *μ*L RT Primer Mix; 4 *μ*L RNase Free dH_2_O; and 10 *μ*L reaction solution from step 1, which were used to reach a total reaction volume of 20 *μ*L. The condition of reverse-transcription (RT) reaction was as follows, 15 min at 37°C and 85°C for 5 s, and stored at 4°C.

All the primers set spanned an intron and the information of primer was collected in Table 1. The PCR reaction of components was combined in a master mix composed of 10 *μ*L SYBR* Premix Ex Taq* II (2x); 0.8 *μ*L PCR Forward Primer (0.4 *μ*M); 0.8 *μ*L PCR Reverse Primer (0.4 *μ*M); 0.4 *μ*L ROX Reference Dye II (50x); 6 *μ*L dH_2_O; 2 *μ*L cDNA.

The real-time quantitative PCR was conducted in ABI 7500 (Applied Biosystems, USA) and the cycling program was set at 1 cycle of predenaturation at 95°C for 30 s and then 40 cycles at 95°C for 15 s, 56°C for 30 s, and 72°C for 31 s. All the real-time PCR experimentation was conducted strictly according to the rules of the MIQE. Quantification of the target cDNAs in all samples was normalized to GAPDH rRNA (Ct_target_ − Ct_GAPDH_ = ΔCt) and the difference in expression for each target cDNA in the investigated groups was expressed to the amount in the water control group (ΔCt_treated_ − ΔCt_control_ = ΔΔCt). Fold changes in target gene expression were determined by taking 2 to the power of this number (2^−ΔΔCt^).

### 2.9. Statistical Analysis

All data were shown as means ± standard deviation, and *n* referred to the number of animals used in each experiment. Pharmacokinetic analysis was done by PK Software DAS 2.0 (Bontz Inc., Beijing, China). All statistical tests were performed using SPSS for windows (SPSS 17.0, Chicago, IL). Comparisons among groups were carried out using one-way analysis of variance (ANOVA) followed by least significant difference (LSD) test for multiple comparisons of observed differences between means. Significance was determined at a probability of *p* < 0.05.

## 3. Results

### 3.1. Pharmacokinetic Studies

**Figure 1:**
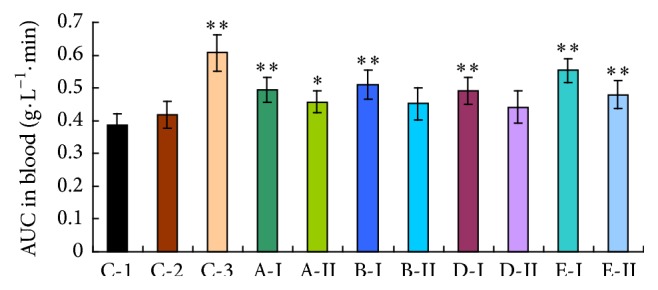
The AUC of PAH in sera after treatment with various investigational samples. C-1: water control group; C-2: 0.5% CMC-Na group; C-3: probenecid group. Higher dosages were expressed by I, and lower dosages were expressed by II. A: Realgar, B: levigated Realgar, D: Cinnabar, and E: HgCl_2_ (full text). The results are expressed as the mean ± s.d.; ^*∗*^ *p* < 0.05; ^*∗∗*^ *p* < 0.01 in comparison with C-1 (LSD test).

The major pharmacokinetic parameters of serum PAH in mice were shown in Table 2. Both 0.5% CMC-Na group and water control group showed no significant differences in each examined pharmacokinetic parameter. For all investigational sample groups, the total apparent volume of distribution (Vd_T_) was observably decreased, total clearance (CL_T_) was remarkably reduced, and the area under the curve (AUC_0–30 min_) was significantly increased (see Figure 1). Elimination half-life (*t*_1/2*β*_) however was markedly prolonged only in HgCl_2_ group.

### 3.2. PAH Accumulation Studies in Kidney

**Figure 2:**
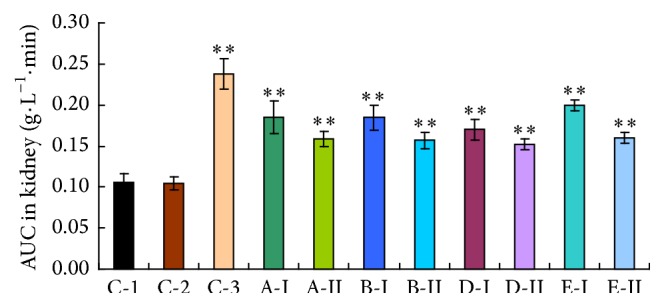
The AUC of PAH in kidney tissue after treatment with various investigational samples. C-1: water control group; C-2: 0.5% CMC-Na group; C-3: probenecid group. Higher dosages were expressed by I, and lower dosages were expressed by II. A: Realgar, B: levigated Realgar, D: Cinnabar, and E: HgCl_2_ (full text). The results are expressed as the mean ± s.d.; ^*∗*^ *p* < 0.05; ^*∗∗*^ *p* < 0.01 in comparison with C-1 (LSD test).

As shown in Figure 2, comparing with C-1 group, the PAH accumulations were not influenced by 0.5% CMC-Na (*p* > 0.05). However, the PAH accumulations in the kidneys of investigational sample groups (A, B, D, and E) were observably increased at all sampling times after i.v. administration of PAH (*p* < 0.01). The accumulations are evidenced by the AUC_0–30 min_ of PAH in kidney tissue for each sample. The distribution profiles in kidney tissue were very similar to those in blood.

### 3.3. PAH Uptake by Mice Renal Slices

**Figure 3:**
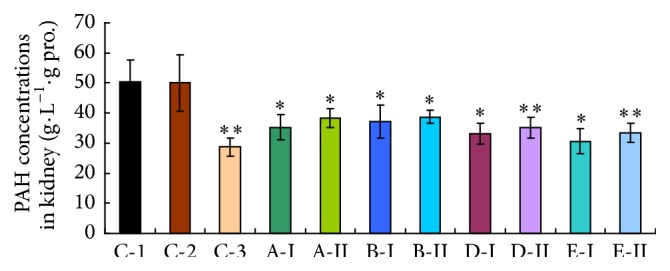
Effect of the investigational samples on PAH uptake by kidney slices of mice after treatment twice a day for continuous five days. C-1: water control group; C-2: 0.5% CMC-Na group; C-3: probenecid group. Higher dosages were expressed by I, and lower dosages were expressed by II. A: Realgar, B: levigated Realgar, D: Cinnabar, and E: HgCl_2_. Each column represents the mean ± s.d. (*n* = 10). ^*∗∗*^ *p* < 0.01, significantly different from C-1/C-2.

As shown in Figure 3, the active uptake of PAH by renal slices was observably inhibited by all the investigational compounds (*p* < 0.01). However, the PAH uptake of the renal slices in 0.5% CMC-Na group (C-2) has no influence compared with control one (*p* > 0.05).

### 3.4. mRNA Expression Levels

**Figure 4:**
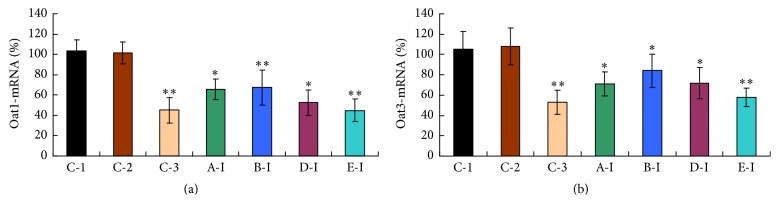
Expression levels of (a) Oat1 and (b) Oat3 mRNA in the kidney tissue of mice after treatment with investigational samples. C-1: water control group; C-2: 0.5% CMC-Na group; C-3: probenecid group. Higher dosages were expressed by I, and lower dosages were expressed by II. A: Realgar, B: levigated Realgar, D: Cinnabar, and E: HgCl_2_. All data were expressed as percentages referred to the corresponding control, and are expressed mean ± s.d. (*n* = 12). Asterisks sign designates significant differences. ^*∗*^ *p* < 0.05 versus C-1/C-2; ^*∗∗*^ *p* < 0.01 versus C-1/C-2.

As shown in Figure 4, Oat1 and Oat3 mRNA levels were not influenced in 0.5% CMC-Na (C-2) group compared with water control (*p* > 0.05). Both Oat1 and Oat3 mRNA levels were clearly downregulated in kidneys from all investigational groups as compared with C-1/C-2 groups (*p* < 0.01).

## 4. Discussion

Arsenic and mercury have been recognized as a hazardous environmental pollutant which is harmful to the plants, animals, and even mammals; people can be easily exposed to them through contaminated water and food [24, 25]. The soluble arsenic (As^3+^) and mercury (Hg^2+^) can be simply absorbed in the gastrointestinal tract and distributed throughout the body. It is worth noting that the oral LD_50_ for Realgar (As) and Cinnabar (Hg) in mice is about 3200 mg/kg and 2678 mg/kg, but the oral LD_50_ for arsenic trioxide (As^3+^), arsenic pentoxide (As^5+^), and divalent mercury (Hg^2+^) in mice is as small as 33 to 39 mg/kg, 112 to 175 mg/kg, and 7 to 10 mg/kg, respectively, dozens of times difference compared with Realgar and Cinnabar [26–28]. As_2_O_3_ and HgCl_2_ are two established nephrotoxicant compounds in mice and rats which dose-dependently affect the membrane transporters (Oat1 and Oat3) of the proximal tubules [29]. At the basolateral membrane of proximal tubular cells, the organic anion transporters (Oat1 and Oat3) mediate the uptake of a number of As^3+^- or Hg^2+^-thiol conjugates from plasma [30]. The ability of these carriers to transport As^3+^ or Hg^2+^ is thought to be dependent upon the conjugation of low molecular weight thiols (such as cysteine and homocysteine) with As^3+^ or Hg^2+^ [31].

According to the literature, divalent mercury is a highly toxic element because of its accumulative and persistent nature in the environment and biota [32]. Cinnabar usually slightly contains about 0.011~2.98% of the divalent mercury [14, 33]. In Cinnabar used in our experiments, however, the content of water soluble mercury was 0.07% and mercuric sulfide was 98.6%, and those are consistent with the reported studies [14, 33].

In the present study, each of two different dosages of Realgar, levigated Realgar (it was processed by levigated courses described in the Chinese Pharmacopoeia), and Cinnabar were designed, respectively, and they were about four times (higher dosage) or equivalent (lower dosage) to the higher dosages of Chinese Pharmacopoeia (according to the calculations of mice and human body surface area at the equivalent to the clinical dosage) [34]. However, the soluble mercury in Cinnabar was almost of equal amounts to the dosage of HgCl_2_ administered in the present study according to the result of AAS determination.

The current PAH-clearance test shows that mice treated with investigational compounds (Realgar, levigated Realgar, Cinnabars, and HgCl_2_) exhibited an obvious decrease in the total volume of distribution (Vd_T_) and a significant increase in the area under the curve (AUC). Nevertheless, the decrease in the total clearance (CL_T_) of PAH might be justified on one hand by the decreased PAH uptake in renal basolateral membranes and on the other hand by the fact that Oat1 and Oat3 mediate the uptake of a number of As^3+^- or Hg^2+^-thiol conjugates [31, 35]. Even the constants *α*, *β*, *k*_12_, *k*_21_, and *k*_10_ (data not shown) were slightly decreased in each group of the tested mice. Based upon this observation, Realgar and Cinnabar may affect pharmacokinetics of PAH by inhibition of renal excretion via Oat1 and Oat3. This leads us to guess that organic anion transporters play an important role not only in the elimination of PAH via the kidney but also in mediating the nephrotoxicity of heavy metals tested in the experiments.

In kidneys, the first step in active secretion is the extraction of organic anions from the peritubular blood plasma by the proximal tubular cells through the basolateral membrane [21]. Probe substrates for, and inhibitors of, specific transporters are desired to evaluate quantitatively* in vivo* functions of transporters in mice. This basolateral uptake of organic anions has been extensively investigated with PAH as the test substrate [36]. However to evaluate the functional activity of Oat1 and Oat3 in renal tubular cells, we measured PAH uptake in mice kidney slices prepared from whole kidney of all investigational groups according to the method of Henderson and Lindup [23]. The results have showed that a significant reduction in PAH uptake via mice renal slices was found, which means the activity of Oat1 and Oat3 was markedly inhibited by the investigational compounds. The differences in PAH uptake indicate that a lower number of functional carrier units exist in renal slices which were made from tested mice; this is also in agreement with the lower activities of Oat1 and Oat3 in renal slices.

AUC is generally considered to be one of the most important PK parameters in the pharmacokinetic study, so the present study majorly concerned the AUC of PAH in blood and in kidney tissue for each tested sample. Compared with the water/CMC control group, both serum AUC_0–30 min_ and kidney AUC_0–30 min_ in each tested sample were all increased (44% to 88% for kidney, 14% to 42% for serum), whereas the ratios of AUC_0–30 min_ for kidney and serum were comparatively constant, around 0.3 (0.27~0.31), which means no significant difference between the groups, indicating that PAH was almost not influenced by the other transporters or metabolic enzymes except OATs. When the blood PAH concentrations were increased, the renal tissue PAH concentrations were also increased in a similar proportion, but the AUC ratio (kidney/serum) was almost the same in each group, ranging from 0.27 to 0.31.

The mice genes of Oat1 and Oat3 are highly homology with human OATs (OAT1, OAT3) [37, 38], which are also highly expressed in the basolateral membranes of renal proximal tubular cells [11, 39]. In this experiment, Oat1 and Oat3 mRNA expressions significantly decreased in the renal cortex of mice in all tested sample groups. The over 20% reduction of Oat1 mRNA expression may be mediated by the arsenic and mercury toxicity-dependent inhibition of the specific genes for Oat1 [9]. The expression of Oat3 mRNA was significantly reduced in kidneys from all investigational samples, indicating a clear downregulation at the amplification level of mRNA happens. However, obvious decrease in Oat1 and Oat3 mRNA levels (40%) was observed in HgCl_2_ group mice and also suggests that the inhibiting effect of HgCl_2_ on OATs may be stronger than Cinnabars in the same dosage. Hence, Oat1 and Oat3 mediate arsenic and mercury compounds' access to the proximal tubule cells; their downregulation mRNA expression might be another defensive mechanism developed by the cell to protect itself against arsenic and mercury injury.

As a conclusion, in consideration of the results mentioned above, we have found that all investigational compounds (Realgar, levigated Realgar, Cinnabars, and HgCl_2_) interfered with the PAH uptake through Oat1 and Oat3 in renal basolateral membrane of proximal tubule. Although the theory of traditional Chinese medicine says preparation procedures (like water grinding) for Realgar and Cinnabar could always reduce the toxicity and enhance the therapeutic effect for them, unfortunately, the water levigated process could not completely delete the water soluble substances contained in Realgar and Cinnabar and they still remarkably regulate the function of organic anion transporters of Oat1 and Oat3.

Drug-drug interactions take place at OAT1/Oat1 and OAT3/Oat3 level may retard the excretion of endo- or exogenous toxic compounds and then cause serious unwanted side effects. We rediscovered that Realgar and Cinnabar which were known as potential carcinogen [40] are dangerous and even processed before use according to theory of Chinese medicine via the experiment. We still need to take with caution and should discern that preparation procedures (like water grinding) for them are probably ineffective in reducing the toxicity of known toxic substances like Realgar and Cinnabar.

## Figures and Tables

**Table 1:** Sequences of the primers of mOat1 and mOat3 for RT-PCR.

Genes	Accession number	Sense primers (5′ to 3′)	Antisense primers (5′ to 3′)	Product size (bp)
mOat1	NM_008766	ATGCCTATCCACACCCGTGC	GGCAAAGCTAGTGGCAAACC	417
mOat3	NM_031194	CAGTCTTCATGGCAGGTATACTGG	CTGTAGCCAGCGCCACTGAG	338
GAPDH	M32599	GGTGAAGGTCGGTGTGAACG	CTCGCTCCTGGAAGATGGTG	233

**Table 2:** Major pharmacokinetic parameters in mice sera after a single dose of PAH.

Groups	*t*_1/2*β*_/min	Vd_T_/L·kg^−1^	CL_T_/mL·min^−1^·kg^−1^	AUC_0–30 min_/g·L^−1^·min
C-1	9.88 ± 0.83	0.423 ± 0.05	53.2 ± 5.3	0.464 ± 0.043
C-2	10.06 ± 0.71	0.428 ± 0.03	51.9 ± 3.4	0.474 ± 0.030
C-3	11.57 ± 1.59^*∗*^	0.302 ± 0.01^*∗∗*^	33.9 ± 2.7^*∗∗*^	0.721 ± 0.053^*∗∗*^
A-I	10.05 ± 1.45	0.331 ± 0.03^*∗∗*^	41.5 ± 3.7^*∗∗*^	0.599 ± 0.045^*∗*^
A-II	10.91 ± 0.86^*∗*^	0.325 ± 0.02^*∗∗*^	43.7 ± 2.6^*∗∗*^	0.555 ± 0.037^*∗∗*^
B-I	11.07 ± 1.50^*∗*^	0.346 ± 0.03^*∗*^	40.5 ± 4.1^*∗∗*^	0.608 ± 0.049^*∗∗*^
B-II	10.13 ± 0.90	0.387 ± 0.04	46.2 ± 5.2	0.539 ± 0.054
D-I	10.83 ± 1.33	0.352 ± 0.03	41.9 ± 3.9^*∗∗*^	0.585 ± 0.045^*∗∗*^
D-II	10.68 ± 0.91	0.378 ± 0.03	46.7 ± 5.0	0.527 ± 0.054
E-I	12.40 ± 2.08^*∗∗*^	0.320 ± 0.02^*∗∗*^	36.6 ± 3.2^*∗∗*^	0.660 ± 0.040^*∗∗*^
E-II	11.89 ± 1.17^*∗∗*^	0.329 ± 0.03^*∗∗*^	41.8 ± 3.7^*∗∗*^	0.579 ± 0.048^*∗∗*^

Note: C-1: water control group; C-2: 0.5% CMC-Na group; C-3: probenecid group. Higher dosages were expressed by I, and lower dosages were expressed by II. A: Realgar, B: levigated Realgar, D: Cinnabar, and E: HgCl_2_ (full text). *t*_1/2*β*_ elimination half-life, CL_T_ total clearance, Vd_T_ total volume of distribution, and AUC area under curve. The results are expressed as the mean ± s.d.; ^*∗*^ *p* < 0.05; ^*∗∗*^ *p* < 0.01 in comparison with C-1 (LSD test).
